# 

**Published:** 1843-11

**Authors:** 


### CONTENTS

OF NO. V.

---

### ORIGINAL COMMUNICATIONS.

### ESSAYS AND CASES.

Art. I.—An Account of the Fever which occurred among the Theological Students of Lane Seminary, during the fall of 1842, and the ensuing winter. By Thomas Carroll, M.D., of Cincinnati. - - - 322

Art. II.—Operation of Lithotrity. By William A. McDowell, M.D., of Louisville. - - - - 364

### SELECTIONS FROM AMERICAN AND FOREIGN JOURNALS.

On the Characters and Structural peculiarities of a Group of Morbid Growths in which Cancerous Affections are included. By Dr. Hodgkin. - - - - 367

Homœopathic Experiments. By Andral. - - - 368

New Mode of Accelerating Labour. By Samuel Staniland. 370

Examination of the Eye in Disease. - - - - - 372

Remarks on some forms of Abdominal Neuralgia depending on Uterine Irritation. By Golding Bird, A.M., M.D., F. L.S., &c . - - - - - - - 376

The Medical Profession- - - - - - - 381

Treatment of Chancre. - - - - - - 383
On the Difference of the Respiratory Movements at Different Ages, etc. - - - - - - 385
Continental Treatment of Neuralgia. - - - - 388
Normal Proportions of the Horse. - - - - 388
Ioduretted Syrup of Sarsaparilla. - - - - 389
New Preservative for Animal Substances. - - - 390
Treatment in Cholera. - - - - - 390
Nervous Headache, &c. - - - - - 390
Treacle (Molasses) and Water for Burns. - - - 390
Case of Poisoning by Lead in Maccouba Snuff. - - 391
Treatment of Varicose Veins. - - - - 393
Herpetic Pruritus. - - - - - - 393
Pruritus of the Vulva. - - - - - 393
Gallic Acid in Menorrhagia. - - - - - 394

ORIGINAL INTELLIGENCE.

Professor Caldwell on Animal Chemistry. - - - 395
Graham's Chemistry, with Notes and Additions, by Robert Bridges, M.D. - - - - - - 396
Operation for Club Foot. - - - - - 397
Health of the South. - - - - - 398
Johnston's Manual of Chemistry. - - - - 400
Death of Dr. Harlan. - - - - - 400
Medical Institute of Louisville. - - - - 400

